# Propofol-Based Total Intravenous Anesthesia is Associated with Better Survival than Desflurane Anesthesia in Epithelial Ovarian Cancer Surgery: A Retrospective Cohort Study

**DOI:** 10.3389/fphar.2021.685265

**Published:** 2021-09-24

**Authors:** Wei-Cheng Tseng, Meei-Shyuan Lee, Ying-Chih Lin, Hou-Chuan Lai, Mu-Hsien Yu, Ke-Li Wu, Zhi-Fu Wu

**Affiliations:** ^1^ Department of Anesthesiology, Tri-Service General Hospital and National Defense Medical Center, Taipei, Taiwan; ^2^ School of Public Health, National Defense Medical Center, Taipei, Taiwan; ^3^ Department of Obstetrics and Gynecology, Tri-Service General Hospital and National Defense Medical Center, Taipei, Taiwan; ^4^ Department of General Medicine, Tri-Service General Hospital and National Defense Medical Center, Taipei, Taiwan; ^5^ Department of Anesthesiology, Kaohsiung Medical University Hospital, Kaohsiung Medical University, Kaohsiung, Taiwan; ^6^ Department of Anesthesiology, Faculty of Medicine, College of Medicine, Kaohsiung Medical University, Kaohsiung, Taiwan

**Keywords:** cancer surgery, desflurane, epithelial ovarian cancer, propofol, survival

## Abstract

**Background:** Previous studies have shown that anesthetic techniques can affect outcomes of cancer surgery. We investigated the association between anesthetic techniques and patient outcomes after elective epithelial ovarian cancer surgery.

**Methods:** This was a retrospective cohort study of patients who received elective open surgery for epithelial ovarian cancer between January 2009 and December 2014. Patients were grouped according to the administration of propofol or desflurane anesthesia. Kaplan–Meier analysis was performed, and survival curves were constructed from the date of surgery to death. Univariate and multivariate Cox regression models were used to compare hazard ratios for death after propensity matching. Subgroup analyses were performed for age, body mass index, preoperative carbohydrate antigen-125 level, International Federation of Gynecology and Obstetrics staging, and operation and anesthesia time.

**Results:** In total, 165 patients (76 deaths, 46.1%) who received desflurane anesthesia and 119 (30 deaths, 25.2%) who received propofol anesthesia were eligible for analysis. After propensity matching, 104 patients were included in each group. In the matched analysis, patients who received propofol anesthesia had better survival with a hazard ratio of 0.52 (95% confidence interval, 0.33–0.81; *p* = 0.005). Subgroup analyses also showed significantly better survival with old age, high body mass index, elevated carbohydrate antigen-125 level, advanced International Federation of Gynecology and Obstetrics stage, and prolonged operation and anesthesia time in the matched propofol group. In addition, patients administered with propofol anesthesia had less postoperative recurrence and metastasis than those administered with desflurane anesthesia in the matched analysis.

**Conclusion:** Propofol anesthesia was associated with better survival in patients who underwent elective epithelial ovarian cancer open surgery. Prospective studies are warranted to evaluate the effects of propofol anesthesia on oncological outcomes in patients with epithelial ovarian cancer.

## Introduction

Ovarian cancer is the seventh most common cancer among women worldwide (Lheureux et al., 2019a; Lheureux et al., 2019b), and epithelial ovarian cancer (EOC) accounts for over 95% of ovarian malignancies (Lheureux et al., 2019a). Because EOC is often diagnosed at an advanced stage, the outcomes of the disease are complicated, making it the most lethal gynecological cancer, with a 5-year survival rate of 46% (Lheureux et al., 2019b). Surgery has been a mainstay of therapy for EOC and allows for accurate surgical staging and therapeutic effects by debulking the disease (Lheureux et al., 2019a). However, surgery-induced stress may lead to immunosuppression and upregulation of adhesion molecules through mechanisms involving inflammation, ischemia-reperfusion injury, sympathetic nervous system activation, and increased cytokine release (Chen et al., 2019). The combination of potential tumor cell dissemination and impaired immune response produces an environment favorable for the development of cancer recurrence and metastasis. Accordingly, there is increasing interest in the impact of the perioperative setting on cancer progression.

Accumulating evidence shows that different anesthetic agents or techniques can influence immune function and tumor development in various pathways (Snyder and Greenberg, 2010; Kim, 2018; Chen et al., 2019). Experimental studies showed that volatile anesthetics (VAs) may alter immunological processes that increase metastatic potential (Shapiro et al., 1981; Moudgil and Singal, 1997; Melamed et al., 2003), whereas propofol seemed to maintain the integrity of immunity and reduce the tendency toward cancer metastasis (Mammoto et al., 2002; Melamed et al., 2003; Kushida et al., 2007). Such effects of volatile and propofol anesthesia were also reported in clinically surgical settings, indicating the superiority of propofol over VAs in cancer surgery (Buckley et al., 2014; Zhang et al., 2014; Liu et al., 2016; Ai and Wang, 2020). In addition, results from retrospective studies reported that propofol-based anesthesia produced better long-term outcomes than VAs-based anesthesia after surgery in different types of cancers (Wigmore et al., 2016; Jun et al., 2017; Wu et al., 2018; Lai et al., 2019a; Lai et al., 2019b; Huang et al., 2020; Lai et al., 2020a; Lai et al., 2020b). However, some studies did not show definite effects of anesthetic agents on cancer immunity and outcomes (Lim et al., 2018; Oh et al., 2018; Huang et al., 2019; Yoo et al., 2019; Grau et al., 2020; Makito et al., 2020). Notably, a recent meta-analysis showed that propofol-based total intravenous anesthesia is generally associated with better overall survival than volatile anesthesia in cancer surgery, especially in patients who received desflurane anesthesia (Chang et al., 2021).

Previous studies have shown that intraoperative use of epidural anesthesia was associated with improved oncological outcomes in patients with ovarian cancer (de Oliveira et al., 2011; Tseng et al., 2018). To the best of our knowledge, there is a retrospective cohort study discussing the impacts of different VAs during anesthesia maintenance on survival outcomes after EOC surgery and concluding that patients with advanced EOC who were administered with desflurane anesthesia experienced a lower rate of disease recurrence and an improved disease-free survival after primary cytoreductive surgery compared with those who were administered with sevoflurane anesthesia (Elias et al., 2015). However, no known study has compared the effects between propofol and VAs on patient outcomes after EOC surgery. We hypothesized that propofol anesthesia was associated with greater overall survival than desflurane anesthesia as our previous studies (Wu et al., 2018; Lai et al., 2019a; Lai et al., 2019b; Huang et al., 2020; Lai et al., 2020a; Lai et al., 2020b). Therefore, we conducted a retrospective analysis to assess the relationship between the type of anesthesia and long-term outcomes after EOC surgery and to identify potential risk factors for mortality.

## Methods

### Study Design and Setting

This retrospective cohort study was conducted at Tri-Service General Hospital (TSGH), Taipei, Taiwan.

### Participants and Data Sources

The ethics committee of TSGH approved this retrospective study and waived the need for informed consent (TSGHIRB No: 2-106-05-132). Relevant information was retrieved from the medical records and electronic database at TSGH for 284 patients with an American Society of Anesthesiologists (ASA) score of II to III who had undergone elective EOC open surgery for International Federation of Gynecology and Obstetrics (FIGO) stage I to IV EOC between January 2009 and December 2014. Patients included for analysis were administered with either propofol anesthesia (*n* = 119) or desflurane anesthesia (*n* = 165), based on the anesthesiologist’s preference.

**FIGURE 1:**
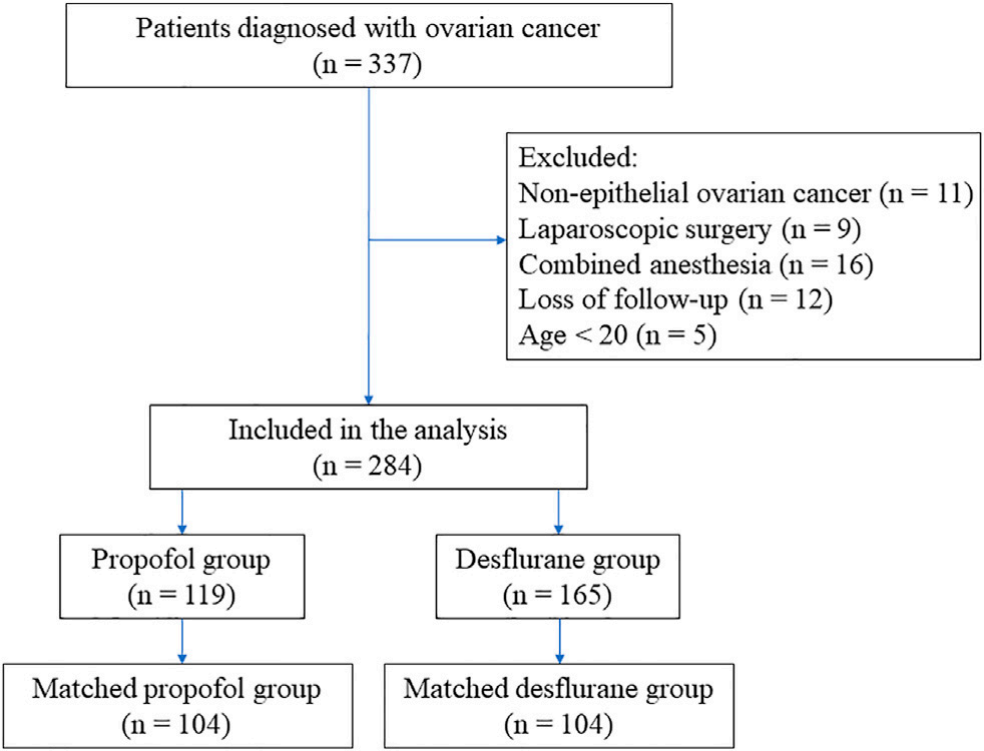
Flow diagram detailing the selection of patients included in the retrospective analysis. Of the patients, 53 were excluded because of combined propofol anesthesia with inhalation anesthesia or regional analgesia, non-epithelial ovarian cancer, incomplete data, age <20 years old, or undergoing laparoscopic surgery.

Exclusion criteria were propofol anesthesia combined with VAs or regional analgesia, laparoscopic surgery, non-EOC, incomplete data, and age under 20 years. Ultimately, 53 cases were excluded from this analysis (Figure 1).

### Anesthetic Technique

No premedication was given before the induction of anesthesia. Standard monitoring, including electrocardiography (lead II), noninvasive blood pressure, pulse oximetry, end-tidal carbon dioxide (EtCO_2_), and direct radial arterial blood pressure measures, was instituted for each patient. Anesthesia was induced with fentanyl, propofol, and cisatracurium or rocuronium in all patients.

In the propofol group, anesthesia was maintained with a target-controlled infusion (TCI) pump (Orchestra^®^ Base Primea, Fresenius Kabi AG, Bad Homburg, Germany) using propofol at an effect-site concentration of 3–4 mcg/ml in a fraction of inspired oxygen (FiO_2_) of 100% oxygen at a flow rate of 300 ml/min. In the desflurane group, the desflurane vaporizer was set between 4 and 10% with 100% oxygen at a flow rate of 300 ml/min in a closed breathing system. Repetitive bolus injections of fentanyl and cisatracurium or rocuronium were administered as necessary during surgery. According to the hemodynamics, the maintenance of anesthesia with desflurane and the effect-site concentration of propofol using a TCI pump were adjusted upward and downward by 0.5–2% and 0.2–0.5 mcg/ml, respectively. The level of EtCO_2_ was maintained at 35–45 mmHg by adjusting the ventilation rate in the volume control model with a tidal volume of 6–8 ml/kg and a maximum airway pressure <30 cmH_2_O. After surgery, all patients were transferred to the post-anesthesia care unit for postoperative observation and care (Wu et al., 2018; Lai et al., 2019a; Lai et al., 2019b; Lai et al., 2020a; Lai et al., 2020b).

### Variables

We retrospectively collected the following patient data: anesthetic technique; time since the earliest included patient serving as a surrogate of the calendar year; calendar period; age at the time of surgery; habitus; underlying disease; menstrual and reproductive factors; FIGO stage and histological grade of the primary tumor; presence of pleural effusion or ascites before surgery; and pretreatment serum level of carbohydrate antigen-125 (CA-125). For pretreatment serum CA-125 values, patients were grouped according to CA-125 values of ≥35 or <35 U/ml because a CA-125 level ≥35 U/ml was associated with poor survival in patients with EOC (Lin et al., 2020).

The Charlson comorbidity index (CCI) of 0 (least comorbidity) to 37 (highest comorbidity) was used to predict the 10-year survival in patients with multiple comorbidities. In addition, preoperative functional status was evaluated in metabolic equivalents (METs), and patients were grouped according to a functional status of ≥4 or <4 METs because perioperative cardiac and long-term risks increased in patients with a capacity of <4 METs during most normal daily activities (Fleisher et al., 2014).

Other data included the ASA physical status score from I (lowest morbidity) to V (highest morbidity); administration of neoadjuvant or adjuvant chemotherapy; need for intraoperative blood transfusion; use of postoperative nonsteroidal anti-inflammatory drugs (NSAIDs); operation and anesthesia time; total propofol dosage including induction dose; grade of surgical complications using the Clavien–Dindo classification scaled from 0 (no complication) to V (death); length of hospital stay; presence of postoperative recurrence or metastasis; and mortality. Postoperative recurrence could be identified by physical examination, radiological evidence, and serum CA-125 monitoring. Concerning levels of serum CA-125, postoperative recurrence was defined by the rise of more than twice the upper limit of normal (35 U/ml) 1) for patients with normal baseline CA-125 levels, or for those whose CA-125 levels had normalized during treatment; and 2) the rise of more than twice nadir value for patients whose CA-125 levels had not normalized. Postoperative metastasis was defined by the new development of 1) pleural effusion with positive cytology; 2) liver or splenic parenchymal metastasis; 3) metastasis to extra-abdominal organs (including inguinal lymph nodes and lymph nodes outside the abdominal cavity); and 4) transmural involvement of intestine, which was not detected before surgery (Lheureux et al., 2019b). Based on causes of death, patients who died at the follow-up period from the date of surgery to December 31, 2019 were recorded as all-cause or cancer-specific mortality. All-cause mortality was defined that patients died at the follow-up period under various causes including cancer-related or not; cancer-specific mortality was defined that patients died only from cancer-related causes at the follow-up period. Because these variables had been shown or posited to affect patient outcomes, they were chosen as potential confounders.

### Statistical Methods

The primary outcome was overall survival compared between the propofol and desflurane groups. The survival time was defined as the interval between the date of surgery and the date of death or December 31, 2019, for patients who were censored. All data were presented as mean ± standard deviation (SD) or number (percentage).

Patient characteristics and mortality rates were compared between the groups treated with different anesthetics using Student’s t-test or chi-squared test. The survival according to the anesthetic technique was depicted visually in a Kaplan–Meier survival curve. The association between the anesthetic technique (propofol or desflurane) and survival was analyzed using the Cox proportional-hazards model with and without adjustment for variables noted previously. Overall survival from the date of surgery grouped according to the anesthetic technique and other variables was compared separately in a univariate Cox model and subsequently in a multivariate Cox regression model. Variables that were significant in the univariate model proceeded to execute the multivariate analysis, but postoperative recurrence and metastasis were excluded to avoid multicollinearity. We also conducted subgroup analyses for age, body mass index (BMI), preoperative CA-125 level, FIGO stages, operation and anesthesia time, and disease progression between the 2 anesthetic techniques.

Propensity score (PS) matching using IBM SPSS Statistics 23.0 (IBM SPSS Inc., Chicago, IL) was applied to select the most similar PSs for preoperative variables (with caliper sets at 0.2 SD of the logit of the PS) across each anesthesia: propofol or desflurane in a 1:1 ratio, ensuring the comparability between propofol and desflurane anesthesia before surgery. Preoperative variables for performing PS matching included time since the earliest included patient; age; BMI; CCI; ASA class; menstrual and reproductive factors; FIGO stage and histological grade; presence of pleural effusion or ascites; preoperative serum level of CA-125; and administration of neoadjuvant chemotherapy. Because calendar period, underlying disease and functional status were highly correlated with time since the earliest included patient, CCI and ASA class, respectively, these variables were excluded to increase the rigorousness of PS matching. Two-tailed *p* values <0.05 were considered statistically significant.

## Results

### Patient and Treatment Characteristics

Patient and treatment characteristics are shown in Table 1. The time since the earliest included patient; calendar period; age; BMI; CCI; underlying disease; ASA score; preoperative functional status; menstrual and reproductive factors; FIGO stage and histological grade of the primary tumor; presence of pleural effusion and ascites before surgery; baseline CA-125 level; administration of neoadjuvant and adjuvant chemotherapy; need for intraoperative blood transfusion; use of postoperative NSAIDs; operation and anesthesia time; grade of surgical complications; and length of hospital stay were not significantly different between the 2 anesthetic techniques (Table 1). Total propofol dosage in the propofol group was significantly more than that in the desflurane group (Table 1). In addition, no patient underwent postoperative radiotherapy.

**TABLE 1:** Patient and treatment characteristics for overall group and matched group after propensity scoring.

Variables	Overall patients			Matched patients			SMD
	Propofol (*n* = 119)	Desflurane (*n* = 165)	*p* value	Propofol (*n* = 104)	Desflurane (*n* = 104)	*p* value	
Time since the earliest included patient (years), mean (SD)	3.28 (1.67)	3.36 (1.83)	0.723	3.33 (1.69)	2.37 (1.52)	<0.001	0.597
Calendar period, *n* (%)			0.054			0.008	0.443
2009–2010	30 (25.2)	45 (27.3)		26 (25.0)	45 (43.3)		
2011–2012	45 (37.8)	41 (24.8)		38 (36.5)	36 (34.6)		
2013–2014	44 (37.0)	79 (47.9)		40 (38.5)	23 (22.1)		
Age (years/o), mean (SD)	53.70 (11.28)	54.41 (12.17)	0.618	53.98 (11.34)	52.82 (12.19)	0.477	0.099
BMI (kg/m^2^), mean (SD)	23.60 (3.88)	23.55 (3.84)	0.927	23.45 (3.62)	23.31 (3.74)	0.784	0.038
Charlson comorbidity index, mean (SD)	3.55 (1.67)	3.84 (1.96)	0.183	3.57 (1.59)	3.45 (1.70)	0.614	0.073
Underlying disease							
Diabetes mellitus	6 (5.0)	18 (10.9)	0.124	6 (5.8)	9 (8.7)	0.592	0.112
Coronary artery disease	8 (6.7)	13 (7.9)	0.891	7 (6.7)	6 (5.8)	1.000	0.037
Stroke	2 (1.7)	3 (1.8)	1.000	2 (1.9)	2 (1.9)	1.000	0.000
Chronic obstructive pulmonary disease	7 (5.9)	6 (3.6)	0.545	7 (6.7)	3 (2.9)	0.331	0.179
Liver disease	4 (3.4)	10 (6.1)	0.448	4 (3.8)	4 (3.8)	1.000	0.000
Peptic ulcer disease	4 (3.4)	11 (6.7)	0.337	4 (3.8)	5 (4.8)	1.000	0.049
ASA class, *n* (%)			0.195			1.000	0.025
II	96 (80.7)	121 (73.3)		83 (79.8)	84 (80.8)		
III	23 (19.3)	44 (26.7)		21 (20.2)	20 (19.2)		
Functional status, *n* (%)			0.195			1.000	0.025
≥4 METs	96 (80.7)	121 (73.3)		83 (79.8)	84 (80.8)		
<4 METs	23 (19.3)	44 (26.7)		21 (20.2)	20 (19.2)		
Menarche, *n* (%)			0.334			0.782	0.076
≥12 years/o	109 (91.6)	157 (95.2)		98 (94.2)	96 (92.3)		
<12 years/o	10 (8.4)	8 (4.8)		6 (5.8)	8 (7.7)		
Menopause, *n* (%)			0.483			0.940	0.048
≤50 years/o	35 (29.4)	41 (24.9)		29 (27.9)	27 (26.0)		
>50 years/o	41 (34.5)	68 (41.2)		38 (36.5)	38 (36.5)		
Not yet	43 (36.1)	56 (33.9)		37 (35.6)	39 (37.5)		
Parity, *n* (%)			0.713			1.000	0.020
0–1	32 (26.9)	40 (24.2)		30 (28.8)	29 (27.9)		
≥2	87 (73.1)	125 (75.8)		74 (71.2)	75 (72.1)		
FIGO stage of primary tumor, *n* (%)			0.103			0.095	0.253
I and II	63 (52.9)	70 (42.4)		54 (51.9)	41 (39.4)		
III and IV	56 (47.1)	95 (57.6)		50 (48.1)	63 (60.6)		
Histological grade of primary tumor, n (%)			0.511			0.988	0.015
I	14 (11.8)	13 (7.9)		10 (9.6)	10 (9.6)		
II	34 (28.6)	46 (27.9)		30 (28.9)	29 (27.9)		
III	71 (59.6)	106 (64.2)		64 (61.5)	65 (62.5)		
Pleural effusion, *n* (%)	21 (17.6)	37 (22.4)	0.403	17 (16.3)	19 (18.3)	0.855	0.053
Ascites, *n* (%)			0.721			0.652	0.085
None to mild	84 (70.6)	112 (67.9)		74 (71.2)	70 (67.3)		
Moderate to massive	35 (29.4)	53 (32.1)		30 (28.8)	34 (32.7)		
Preoperative CA-125 level, n (%)			0.106			1.000	0.030
≥35 U/ml	98 (82.4)	148 (89.7)		90 (86.5)	91 (87.5)		
<35 U/ml	21 (17.6)	17 (10.3)		14 (13.5)	13 (12.5)		
Neoadjuvant chemotherapy, *n* (%)	7 (5.9)	4 (2.4)	0.239	2 (1.9)	3 (2.9)	1.000	0.065
Adjuvant chemotherapy, *n* (%)	106 (89.1)	154 (93.3)	0.291	94 (90.4)	97 (93.3)	0.613	NA
Intraoperative transfusion, *n* (%)	57 (47.9)	88 (53.3)	0.433	50 (48.1)	57 (54.8)	0.405	NA
Postoperative NSAID, *n* (%)	28 (23.5)	42 (25.5)	0.817	26 (25.0)	27 (26.0)	1.000	NA
Operation time (min), mean (SD)	205.64 (69.24)	214.28 (81.85)	0.350	208.01 (70.38)	210.20 (84.88)	0.840	NA
Anesthesia time (min), mean (SD)	230.13 (69.34)	240.16 (81.39)	0.277	232.52 (70.40)	235.84 (84.50)	0.759	NA
Propofol dosage (mg), mean (SD)	1234.61 (347.68)	116.04 (20.09)	<0.001	1243.26 (349.61)	114.87 (18.99)	<0.001	NA
Grade of surgical complications, *n* (%)			0.849			0.762	NA
0	47 (39.5)	59 (35.8)		41 (39.4)	36 (34.6)		
I	14 (11.8)	17 (10.3)		12 (11.5)	10 (9.6)		
II	54 (45.4)	82 (49.7)		47 (45.2)	52 (50.0)		
III	4 (3.3)	7 (4.2)		4 (3.8)	6 (5.8)		
Length of hospital stay (days), mean (SD)	10.73 (7.09)	11.84 (6.52)	0.173	11.09 (7.30)	11.53 (6.54)	0.646	NA
Postoperative recurrence, *n* (%)	43 (36.1)	100 (60.6)	<0.001	42 (40.4)	64 (61.5)	0.004	NA
Postoperative metastasis, *n* (%)	27 (22.7)	68 (41.2)	0.002	26 (25.0)	43 (41.3)	0.018	NA
All-cause mortality, *n* (%)	30 (25.2)	76 (46.1)	0.001	29 (27.9)	50 (48.1)	0.004	NA
Cancer-specific mortality, *n* (%)	27 (22.7)	73 (44.2)	<0.001	26 (25.0)	49 (47.1)	0.001	NA

Data shown as mean ± SD or *n* (%). Grade of surgical complications: Clavien–Dindo classification. ASA, American Society of Anesthesiologists; BMI, body mass index; FIGO, International Federation of Gynecology and Obstetrics; MET, metabolic equivalent; NA, not applicable; NSAID, nonsteroidal anti-inflammatory drug; SD, standard deviation; SMD, standardized mean difference.

The PS matching is an essential statistical method to minimize the effect of confounding in observational studies (Austin et al., 2018). Therefore, we used the PS from the logistic regression to adjust the baseline characteristics and the choice of treatment between the 2 anesthetic techniques. Altogether, 104 pairs were formed after matching. Patient characteristics and treatment factors of EOC were not significantly different between the matched groups except for time since the earliest included patient, calendar period and total propofol dosage (Table 1).

**FIGURE 2:**
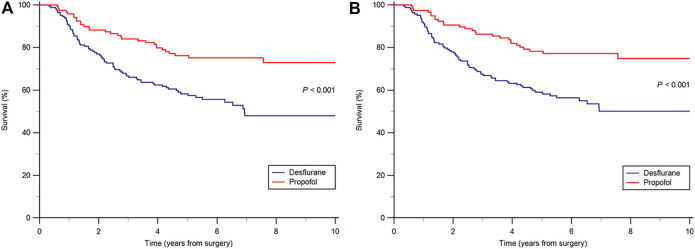
**(A)** Overall **(B)** cancer-specific survival curves from the date of surgery by anesthesia type.

**FIGURE 3:**
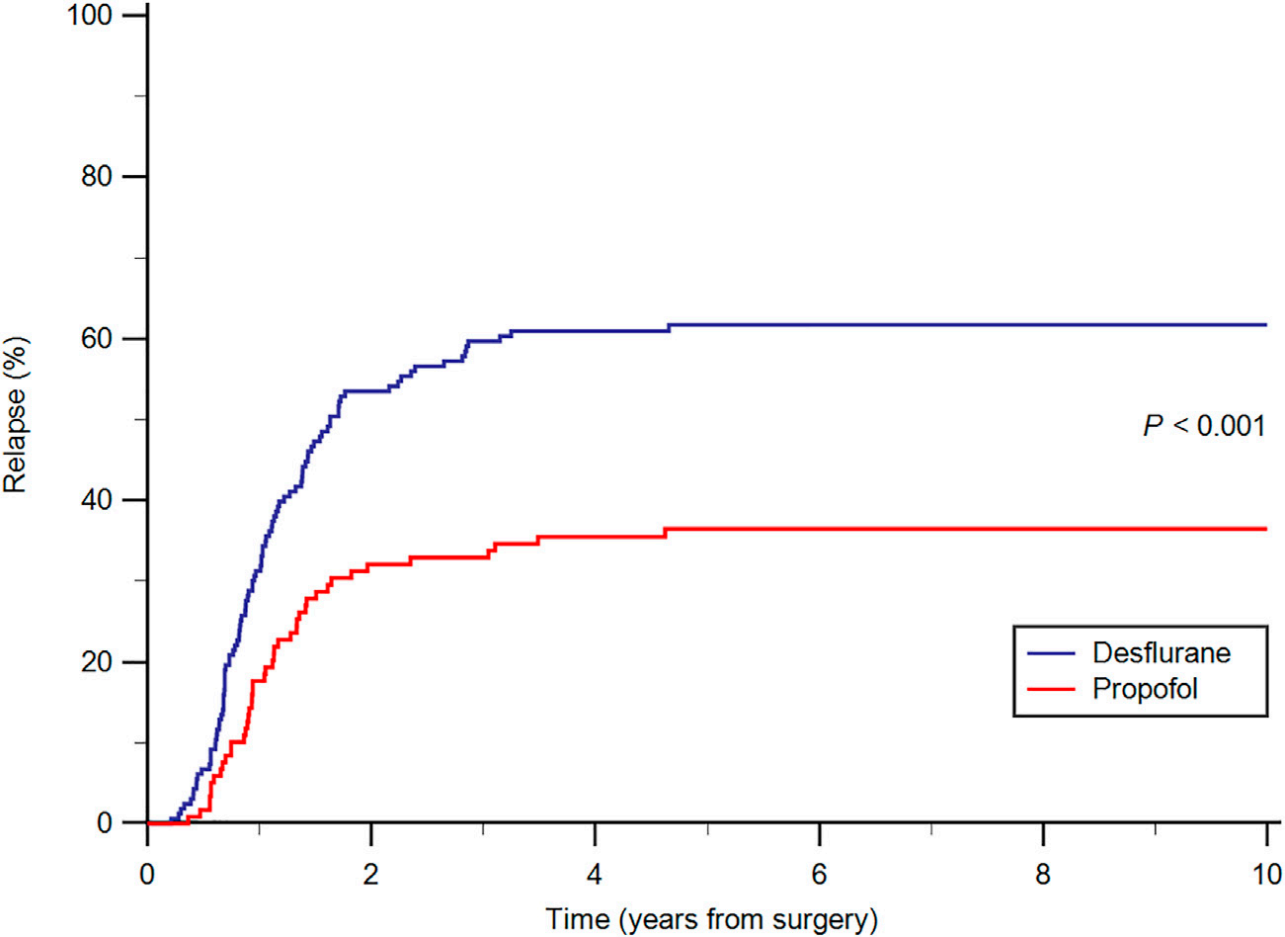
Cumulative relapse curves from the date of surgery by anesthesia type.

A greater percentage of patients in the desflurane group (60.6%) developed postoperative recurrence compared with the propofol group (36.1%; *p <* 0.001). The incidence of postoperative metastasis was also significantly higher in the desflurane group (41.2%) than in the propofol group (22.7%; *p* = 0.002) during follow-up. The all-cause mortality rate was significantly lower in the propofol group (25.2%) than in the desflurane group (46.1%; *p* = 0.001) during follow-up. Furthermore, the cancer-specific mortality rate was significantly lower in the propofol group (22.7%) than in the desflurane group (44.2%; *p* < 0.001) during follow-up. After PS matching, results were consistent between the 2 anesthetic techniques (Table 1). The median follow-up period was 5.86 years for the propofol group and 4.63 years for the desflurane group. Kaplan–Meier survival curves for the 2 anesthetic techniques are shown in Figures 2A,B. In addition, the cumulative incidence of cancer relapse is shown in Figure 3.

### Risks of Overall Mortality

The risk of overall mortality associated with the administration of propofol and desflurane anesthesia during EOC open surgery is shown in Table 2. Patients who received propofol anesthesia had better overall survival than those who received desflurane anesthesia [overall survival, 74.8 versus 53.9%, respectively; hazard ratio (HR), 0.46; 95% confidence interval (CI), 0.30–0.70; *p* < 0.001]. In the multivariate model after adjustment for age at the time of surgery; CCI; ASA score; age at the time of menopause; FIGO stage; histological grade; presence of pleural effusion and ascites before surgery; preoperative CA-125 level; intraoperative blood transfusion; and grade of surgical complications, patients in the propofol group were also associated with improved overall survival than those in the desflurane group (HR, 0.53; 95% CI, 0.34–0.82; *p* = 0.004). Four other variables that significantly increased the mortality risk after the multivariate analysis were menopause at older age (>50 years old; *p* = 0.010), advanced FIGO stage (*p <* 0.001), moderate to massive ascites (*p* = 0.044), and higher baseline CA-125 level (*p* = 0.046) (Table 2).

**TABLE 2:** Cox proportional hazards regression for mortality: univariate and multivariate models for overall patients.

Variables	Univariate		Multivariate	
	HR (95% CI)	*p* value	HR (95% CI)	*p* value
Anesthesia, propofol (ref: desflurane)	0.46 (0.30–0.70)	<0.001	0.53 (0.34–0.82)	0.004
Time since the earliest included patient (years)	0.97 (0.87–1.08)	0.539		
Age (ref: <40)				
40–59 years/o	1.30 (0.64–2.63)	0.473	0.90 (0.39–2.05)	0.798
≧60 years/o	2.18 (1.06–4.46)	0.034	0.74 (0.27–2.01)	0.558
BMI (ref: <24)				
≧24 kg/m^2^	1.09 (0.74–1.60)	0.671		
Charlson comorbidity index	1.27 (1.17–1.38)	<0.001	1.12 (0.99–1.26)	0.073
ASA, III (ref: II)	2.03 (1.36–3.02)	<0.001	0.76 (0.44–1.30)	0.310
Menarche, <12 years/o (ref: ≧12)	0.90 (0.40–2.06)	0.810		
Menopause (ref: ≦50)				
>50 years/o	1.81 (1.12–2.93)	0.015	1.96 (1.18–3.28)	0.010
Not yet	0.84 (0.48–1.45)	0.532	1.36 (0.69–2.65)	0.376
Parity, 0–1 (ref: ≧2)	0.99 (0.64–1.55)	0.972		
FIGO stage, III and IV (ref: I and II)	7.53 (4.35–13.0)	<0.001	4.94 (2.58–9.47)	<0.001
Histological grade (ref: I)				
II	4.51 (1.06–19.1)	0.041	1.83 (0.41–8.25)	0.429
III	7.94 (1.95–32.3)	0.004	2.30 (0.52–10.1)	0.273
Pleural effusion (ref: no)	2.74 (1.84–4.08)	<0.001	0.94 (0.55–1.61)	0.819
Ascites, moderate to massive (ref: none to mild)	3.43 (2.33–5.03)	<0.001	1.68 (1.01–2.79)	0.044
Preoperative CA-125, ≧35 U/ml (ref: <35)	6.77 (2.15–21.3)	0.001	3.57 (1.02–12.4)	0.046
Neoadjuvant chemotherapy (ref: no)	1.12 (0.46–2.76)	0.799		
Operation time (min)	1.00 (1.00–1.01)	0.063		
Anesthesia time (min)	1.00 (1.00–1.01)	0.072		
Intraoperative transfusion (ref: no)	1.70 (1.15–2.51)	0.008	0.09 (0.01–1.06)	0.056
Postoperative NSAID (ref: no)	0.98 (0.63–1.53)	0.943		
Grade of surgical complications (ref: 0)				
I	0.55 (0.23–1.32)	0.182	0.77 (0.31–1.93)	0.577
II	1.57 (1.03–2.38)	0.036	7.11 (0.61–83.3)	0.118
III	1.10 (0.39–3.11)	0.852	4.60 (0.52–40.3)	0.169
Postoperative recurrence (ref: no)	32.9 (13.4–81.1)	<0.001	NA	NA
Postoperative metastasis (ref: no)	8.52 (5.54–13.1)	<0.001	NA	NA

Hazard ratios in the multivariate analyses were adjusted by those variables having significance in the univariate analyses except for postoperative recurrence and metastasis. ASA, American Society of Anesthesiologists; BMI, body mass index; FIGO, International Federation of Gynecology and Obstetrics; HR, hazard ratio; NA, not applicable; NSAID, nonsteroid anti-inflammatory drug.

### Subgroup Analyses

The subgroup analyses for age, BMI, preoperative CA-125 level, FIGO stages, operation and anesthesia time, and disease progression are shown in Table 3. There was no interaction effect between the type of anesthesia and these factors on survival. All analyses were stratified based on age groups, BMI categories, serum CA-125 levels, different FIGO stages, and operation and anesthesia time.

**TABLE 3:** Subgroup analyses for age, BMI, CA-125 level, FIGO stage, operation and anesthesia time, and disease progression.

Stratified variable	Anesthesia	Crude HR (95%CI)	*p* value	*p* value (interaction)	PS-matched HR (95%CI)	*p* value	PS-adjusted HR^a^ (95% CI)	*p* value	PS-adjusted HR^b^ (95% CI)	*p* value
Non-stratified										
	Desflurane	1.00			1.00		1.00		1.00	
	Propofol	0.46	<0.001		0.52	0.005	0.54	0.011	0.48	0.004
		(0.30–0.70)			(0.33–0.81)		(0.34–0.87)		(0.30–0.79)	
Age				0.756						
<40 years/o	Desflurane	1.00			1.00		1.00		1.00	
	Propofol	0.67	0.570		0.68	0.584	0.78	0.728	0.71	0.650
		(0.16–2.71)			(0.17–2.74)		(0.19–3.23)		(0.16–3.12)	
40–59 years/o	Desflurane	1.00			1.00		1.00		1.00	
	Propofol	0.48	0.054		0.59	0.111	0.67	0.265	0.54	0.140
		(0.27–1.06)			(0.30–1.13)		(0.34–1.35)		(0.24–1.22)	
≥60 years/o	Desflurane	1.00			1.00		1.00		1.00	
	Propofol	0.39	0.007		0.37	0.008	0.41	0.020	0.35	0.006
		(0.19–0.77)			(0.18–0.77)		(0.19–0.87)		(0.16–0.74)	
BMI				0.179						
<24 kg/m^2^	Desflurane	1.00			1.00		1.00		1.00	
	Propofol	0.58	0.051		0.66	0.165	0.68	0.201	0.62	0.127
		(0.34–1.01)			(0.36–1.19)		(0.37–1.23)		(0.34–1.15)	
≧24 kg/m^2^	Desflurane	1.00			1.00		1.00		1.00	
	Propofol	0.33	0.001		0.38	0.008	0.40	0.020	0.30	0.006
		(0.17–0.65)			(0.18–0.78)		(0.19–0.87)		(0.12–0.70)	
CA-125				0.323						
<35 U/ml	Desflurane	1.00			1.00		1.00		1.00	
	Propofol	1.70	0.475		2.31	0.664	2.87	0.969	2.77	0.965
		(0.15–18.8)			(0.34–21.1)		(0.61–28.3)		(0.56–27.4)	
≧35 U/ml	Desflurane	1.00			1.00		1.00		1.00	
	Propofol	0.46	<0.001		0.47	0.001	0.49	0.004	0.46	0.002
		(0.30–0.71)			(0.29–0.74)		(0.30–0.80)		(0.28–0.76)	
FIGO stage				0.582						
I and II	Desflurane	1.00			1.00		1.00		1.00	
	Propofol	0.37	0.089		0.53	0.319	0.52	0.328	0.53	0.397
		(0.12–1.16)			(0.15–1.86)		(0.14–1.91)		(0.13–2.28)	
III and IV	Desflurane	1.00			1.00		1.00		1.00	
	Propofol	0.53	0.006		0.60	0.042	0.61	0.048	0.59	0.047
		(0.33–0.83)			(0.37–0.96)		(0.37–0.98)		(0.34–0.98)	
Operation time				0.162						
<180 min	Desflurane	1.00			1.00		1.00		1.00	
	Propofol	0.68	0.312		0.68	0.312	0.72	0.404	0.60	0.223
		(0.33–1.43)			(0.32–1.44)		(0.33–1.56)		(0.26–1.37)	
≧180 min	Desflurane	1.00			1.00		1.00		1.00	
	Propofol	0.37	<0.001		0.43	0.004	0.44	0.007	0.41	0.005
		(0.22–0.63)			(0.24–0.76)		(0.24–0.80)		(0.22–0.77)	
Anesthesia time				0.355						
<180 min	Desflurane	1.00			1.00		1.00		1.00	
	Propofol	0.64	0.302		0.70	0.428	0.72	0.488	0.47	0.186
		(0.27–1.50)			(0.28–1.70)		(0.29–1.82)		(0.15–1.44)	
≧180 min	Desflurane	1.00			1.00		1.00		1.00	
	Propofol	0.41	<0.001		0.46	0.005	0.49	0.011	0.45	0.006
		(0.25–0.67)			(0.27–0.79)		(0.29–0.85)		(0.25–0.80)	
Disease progression										
Postoperative recurrence	Desflurane	1.00			1.00		1.00		1.00	
	Propofol	0.47	<0.001		0.53	0.001	0.55	0.003	0.51	0.001
		(0.33–0.68)			(0.36–0.78)		(0.37–0.81)		(0.34–0.77)	
Postoperative metastasis	Desflurane	1.00			1.00		1.00		1.00	
	Propofol	0.46	0.001		0.53	0.010	0.53	0.012	0.46	0.004
		(0.30–0.72)			(0.32–0.86)		(0.32–0.87)		(0.28–0.78)	
Postoperative recurrence + metastasis	Desflurane	1.00			1.00		1.00		1.00	
	Propofol	0.47	0.001		0.52	0.010	0.53	0.012	0.47	0.004
		(0.30–0.74)			(0.32–0.86)		(0.32–0.87)		(0.28–0.78)	

BMI, body mass index; CA-125, carbohydrate antigen-125; CI, confidence interval; FIGO, International Federation of Gynecology and Obstetrics; HR, hazard ratio; PS, propensity score.

a Adjusted by time since the earliest included patient.

b Adjusted by time since the earliest included patient, operation and anesthesia time.

### Age

Elderly patients who received propofol anesthesia had better survival than those who received desflurane anesthesia. For patients with an age of <40 years old, the crude HR was 0.67 (95% CI, 0.16–2.71; *p* = 0.570), and the PS-matched HR was 0.68 (95% CI, 0.17–2.74; *p* = 0.584). For patients with an age of 40–59 years old, the crude HR was 0.48 (95% CI, 0.27–1.06; *p* = 0.054), and the PS-matched HR was 0.59 (95% CI, 0.30–1.13; *p* = 0.111). For patients with an age of ≥60 years old, the crude HR was 0.39 (95% CI, 0.19–0.77; *p* = 0.007), and the PS-matched HR was 0.37 (95% CI, 0.18–0.77; *p* = 0.008) (Table 3).

### Body Mass Index

Patients with overweight and obesity who received propofol anesthesia had better survival than those who received desflurane anesthesia. For patients with a BMI of <24 kg/m^2^, the crude HR was 0.58 (95% CI, 0.34–1.01; *p* = 0.051), and the PS-matched HR was 0.66 (95% CI, 0.36–1.19; *p* = 0.165). For patients with a BMI of ≥24 kg/m^2^, the crude HR was 0.33 (95% CI, 0.17–0.65; *p* = 0.001), and the PS-matched HR was 0.38 (95% CI, 0.18–0.78; *p* = 0.008) (Table 3).

### Carbohydrate Antigen-125 Level

Patients with an elevated preoperative CA-125 level who received propofol anesthesia had better survival than those who received desflurane anesthesia. For patients with a CA-125 level of <35 U/ml, the crude HR was 1.70 (95% CI, 0.15–18.8; *p* = 0.475), and the PS-matched HR was 2.31 (95% CI, 0.34–21.1; *p* = 0.664). For patients with a CA-125 level of ≥35 U/ml, the crude HR was 0.46 (95% CI, 0.30–0.71; *p* < 0.001), and the PS-matched HR was 0.47 (95% CI, 0.29–0.74; *p* = 0.001) (Table 3).

### International Federation of Gynecology and Obstetrics Stage

Patients with an advanced FIGO stage who received propofol anesthesia had better survival than those who received desflurane anesthesia. For patients with an early FIGO stage (I and II), the crude HR was 0.37 (95% CI, 0.12–1.16; *p* = 0.089), and the PS-matched HR was 0.53 (95% CI, 0.15–1.86; *p* = 0.319). For patients with a late FIGO stage (III and IV), the crude HR was 0.53 (95% CI, 0.33–0.83; *p* = 0.006), and the PS-matched HR was 0.60 (95% CI, 0.37–0.96; *p* = 0.042) (Table 3).

### Operation Time

Patients with prolonged operation time who received propofol anesthesia had better survival than those who received desflurane anesthesia. For patients with operation time of <180 min, the crude HR was 0.68 (95% CI, 0.33–1.43; *p* = 0.312), and the PS-matched HR was 0.68 (95% CI, 0.32–1.44; *p* = 0.312). For patients with operation time of ≥180 min, the crude HR was 0.37 (95% CI, 0.22–0.63; *p* < 0.001), and the PS-matched HR was 0.43 (95% CI, 0.24–0.76; *p* = 0.004) (Table 3).

### Anesthesia Time

Patients with prolonged anesthesia time who received propofol anesthesia had better survival than those who received desflurane anesthesia. For patients with anesthesia time of <180 min, the crude HR was 0.64 (95% CI, 0.27–1.50; *p* = 0.302), and the PS-matched HR was 0.70 (95% CI, 0.28–1.70; *p* = 0.428). For patients with anesthesia time of ≥180 min, the crude HR was 0.41 (95% CI, 0.25–0.67; *p* < 0.001), and the PS-matched HR was 0.46 (95% CI, 0.27–0.79; *p* = 0.005) (Table 3).

### Disease Progression

Patients who received propofol anesthesia had less postoperative recurrence than those who received desflurane anesthesia. The crude HR was 0.47 (95% CI, 0.33–0.68; *p* < 0.001), and the PS-matched HR was 0.53 (95% CI, 0.36–0.78; *p* = 0.001). Patients who received propofol anesthesia had less postoperative metastasis than those who received desflurane anesthesia. The crude HR was 0.46 (95% CI, 0.30–0.72; *p* = 0.001), and the PS-matched HR was 0.53 (95% CI, 0.32–0.86; *p* = 0.010). Patients who received propofol anesthesia had less postoperative recurrence and metastasis than those who received desflurane anesthesia. The crude HR was 0.47 (95% CI, 0.30–0.74; *p* = 0.001), and the PS-matched HR was 0.52 (95% CI, 0.32–0.86; *p* = 0.010) (Table 3).

Due to the significant difference in the time since the earliest included patient between the two groups after PS matching, we adjusted the PS-matched HRs of above-mentioned subgroups by the variable, and the results were consistent with those without adjustment. Concerning the potential impacts of operation and anesthesia time, we also adjusted the PS-matched HRs of above-mentioned subgroups by the time since the earliest included patient, operation and anesthesia time, and found that the results were similar to those without adjustment (Table 3).

In summary, propofol anesthesia was associated with better survival outcomes in EOC patients with old age, high BMI, elevated CA-125 level, advanced FIGO stage, and prolonged operation and anesthesia time, which may imply its protective effects in patients with high risks or receiving complex surgery. In addition, patients who received desflurane anesthesia had poor disease progression than those who received propofol anesthesia.

## Discussion

The main finding in this study was that propofol anesthesia for EOC open surgery improved survival and reduced rates of postoperative recurrence and metastasis compared with desflurane anesthesia. These results were consistent with findings from previous studies that propofol anesthesia was associated with better outcomes compared with volatile anesthesia in some solid cancers (Wigmore et al., 2016; Jun et al., 2017; Wu et al., 2018; Lai et al., 2019a; Lai et al., 2019b; Huang et al., 2020; Lai et al., 2020a; Lai et al., 2020b). Nevertheless, there were retrospective studies reporting insignificant differences in survival between propofol and VAs in surgery for lung, breast, and digestive tract cancers as well as for glioblastoma (Oh et al., 2018; Huang et al., 2019; Yoo et al., 2019; Grau et al., 2020; Makito et al., 2020). As a result, the effects of anesthetic techniques on oncological outcomes from available data are still inconclusive.

Surgical resection is the mainstay of cancer treatment for potentially removable solid tumors. However, tumor cells may disseminate into the vascular and lymphatic systems during surgery and subsequently migrate to distant organs and initiate tumor regrowth and recurrence (Kim, 2018; Chen et al., 2019). Unlike for many cancers, survival rates for ovarian cancer have changed modestly for decades despite advances in screening, surgery, and treatment methods (Lheureux et al., 2019a). In addition, recurrence develops in approximately 75% of women who present with advanced disease (Lheureux et al., 2019b). Because postoperative recurrence and metastasis play important roles in survival and prognosis, discovering how to improve overall survival by reducing the incidence of relapse is requisite. The likelihood of tumor metastasis depends on the balance between the metastatic potential of the tumor and the anti-metastatic host defenses, of which cell-mediated immunity and natural killer cell function in particular are critical components (Snyder and Greenberg, 2010). Growing evidence from animal and human cancer cell line studies has shown that various anesthetics can affect the immune system in different ways and may therefore influence cancer outcomes (Shapiro et al., 1981; Moudgil and Singal, 1997; Mammoto et al., 2002; Melamed et al., 2003; Kushida et al., 2007).

In this study, we found a 48% lower mortality rate with propofol than with desflurane anesthesia in patients after open surgery for EOC. Moreover, propofol anesthesia was also shown to be associated with a lower incidence of postoperative recurrence and metastasis compared with desflurane anesthesia for patients with EOC, comparable with results in patients undergoing hepatocellular carcinoma; intrahepatic cholangiocarcinoma; and colon, prostate, pancreatic, and gastric cancer surgery (Wu et al., 2018; Lai et al., 2019a; Lai et al., 2019b; Huang et al., 2020; Lai et al., 2020a; Lai et al., 2020b). Elias and colleagues (Elias et al., 2015) compared cancer outcomes in patients with advanced EOC who received different VAs and reported the superiority of desflurane over sevoflurane anesthesia. However, no known study has compared the effects of propofol-based versus VAs-based anesthesia on patient outcomes after surgery for EOC. Although our results suggest a potential effect of anesthetics in humans, but it seems biologically implausible that something as complicated as cancer can be reduced by almost a-factor-of-two simply by anesthetic selection. Our results may overestimate the true treatment effect, which is common in retrospective studies. In addition, by contrast with propofol, VAs have very slow terminal elimination from the vessel-rich group and even slower elimination from the whole body, especially in lengthy anesthesia (Lockwood, 2010). Thus, the actual time interval that VAs act in cancer cell biology may be longer than the recorded anesthesia time. Of course, further investigations are warranted to determine the effects of anesthetic techniques on EOC recurrence and metastasis.

Regarding clinicopathological parameters associated with overall survival of patients with EOC, 4 other prognostic factors, including late menopause, advanced FIGO stage, moderate to massive ascites and elevated preoperative CA-125 level, were identified. This study showed that menopause at late age was associated with poor survival after EOC surgery. The finding may indicate at least a middle age (>50 years old) at the time of diagnosis for patients in this population. However, additional research is needed to determine the impact of late menopause on survival. We also found that a higher FIGO stage was associated with poor survival after open surgery for patients with EOC, as noted previously (Fu et al., 2014). Large volume of ascites at initial diagnosis was regarded as another significant factor related with worse oncological outcomes, which may be attributed to the reduced likelihood for complete resection of tumor (Szender et al., 2017). In addition, a higher preoperative CA-125 level was associated with poor survival for patients undergoing EOC surgery, which was consistent with findings from a previous study (Lin et al., 2020).

Laboratory data from human EOC cell lines support the influence of propofol on the behavior of EOC cells through different pathways (Wang et al., 2013; Su et al., 2014; Huang et al., 2016; Sun et al., 2020; Zeng et al., 2020). Using human EOC cell lines, Zeng et al. (2020) showed that propofol inhibited the proliferation and metastasis of EOC cells by enhancing miR-125a-5p, which targeted lin-28 homologue B. Sun et al. (2020) found that propofol could downregulate miR-374a and modulate the forkhead box O1 pathway to reduce the proliferation and cisplatin resistance in EOC cells. Similarly, Huang et al. (2016) reported that propofol hampered the invasion and proliferation of EOC cells via upregulating miR-9 and suppressing NF-kB activation and its downstream matrix metalloproteinase 9 expression. Su et al. (2014) also reported that propofol facilitated the apoptosis of EOC cells through upregulating miR-let-7i. In addition, Wang et al. (2013) suggested that propofol impeded the invasion and metastasis and enhanced the paclitaxel-induced apoptosis in EOC cells through the suppression of the slug expression. Taken together, these findings suggest that propofol induces anti-tumor activity and may be an effective anesthetic agent for use in EOC surgery.

Research on the impacts of VAs on EOC cell biology is limited. Iwasaki et al. (2016) have reported that VAs including isoflurane, sevoflurane and desflurane enhanced the metastatic potential in EOC cells through the increased cellular signaling of chemokine receptor 2. Luo et al. (2015) suggested that isoflurane exposure significantly increased the expression of insulin-like growth factor 1 and its receptor, contributing to cell cycle progression and cell proliferation in EOC cells. A recent study also concluded that sevoflurane and desflurane enhanced cell proliferation and migration of EOC cells via the downregulation of miR-210 and miR-138 (Ishikawa et al., 2021). These studies suggest that VAs including desflurane may enhance the malignant potential of EOC cells. However, there was a previous report showing that sevoflurane could suppress the viability, cell cycle and progression and induce the apoptosis of EOC cells by downregulating stanniocalcin 1 (Zhang et al., 2019). Because of conflicting results, further studies are warranted to clarify the impacts of different VAs on EOC cell biology.

Hypoxia, one of the hallmarks of cancer, is caused by an insufficient oxygen supply, mostly due to a chaotic tumor microcirculation. Solid tumors generally exhibit hypoxia, which is a powerful stimulus for tumor angiogenesis and cancer metastasis; moreover, the hypoxia status of cancer cells may affect the cellular expression program and lead to the resistance to radiotherapy and chemotherapy (Han et al., 2019). Therefore, adaptation of tumor cells to a hypoxic environment may be associated with poor prognosis. Recently, hypoxia-inducible factors (HIFs) have been identified as key regulators of the response to hypoxic stress and are widely discussed. Previous studies have shown that HIF-1α overexpression in ovarian cancer was associated with poor overall survival (Shimogai et al., 2008; Braicu et al., 2014). As for the impacts of anesthetics on the expression of HIF-1α, volatile anesthetics generally upregulated HIF-1α, and propofol could inhibit HIF-1α activation (Kim, 2018). Although no study has been conducted to discuss the effects of anesthetics on the expression of HIFs in EOC cells, propofol anesthesia probably has beneficial effects on the expression of HIFs and subsequently provides better outcomes based on our results.

In addition to cellular signaling processes, the effect of anesthetic agents on components of the immune system is also an important pathway to determine tumor development. Generally, propofol provides its protective effects by increasing cytotoxic T-lymphocyte activity, decreasing pro-inflammatory cytokines, and inhibiting cyclooxygenase-2 and prostaglandin E_2_ functions; in contrast, VAs have been shown to suppress nature killer cell cytotoxicity, induce T-lymphocyte apoptosis, and decrease the T-helper 1/2 ratio (Kim, 2018). The divergent effects on immune function between propofol and VAs may affect the level of surgery-induced immunosuppression and subsequent tumorigenesis. Therefore, in the present study, the mechanism of anesthetic agents contributing to the progression of EOC cells is mainly proposed by directly affecting signaling pathways of tumor cells and indirectly influencing neuroendocrine and immune function.

There were some limitations in this study. First, because this was a retrospective single-center observational study, our findings could not determine the causal relationship between anesthetics and oncological outcomes after EOC surgery; thus, it should be only deemed as hypothesis-generating. Second, the study was retrospective, and patients were not randomly allocated. We conducted PS matching to minimize confounding in this observational study (Austin et al., 2018). However, the small groups for PS matching may influence the reliability of the statistical significance in our study. Fortunately, regardless of the analytic approaches, the point estimation and significance of relative risk of propofol versus desflurane were consistent. Third, although we performed the multivariate analysis and PS matching analysis with many variables to obtain reliable results and valuable information, we could not exclude some unmeasured confounding factors that may be responsible for the result. Fourth, therapeutic methods for EOC patients have evolved over time, which may result in improved outcomes. Because detailed information about surgical techniques and cancer care were not available, we could not completely exclude the possibility that advances in cancer care and surgical techniques may influence survival outcomes. Fifth, there was a lack of data on the levels of immune cells and biomarkers in our study, so we could not confirm the definite relationship between anesthetics, immune and transcriptional factors, and the aggressiveness of the disease. Sixth, different VAs may have distinctive effects on EOC. We only included desflurane in our analysis because it is the most frequently used VA in our hospital. Seventh, we analyzed only the diagnosis of EOC accounting for the majority of ovarian malignancies (Lheureux et al., 2019a), and did not refine the histologic subtypes due to incomplete data. Eighth, we excluded EOC patients undergoing laparoscopic surgery (*n* = 9) to increase the consistency of patient characteristics, although there was no significant difference in oncological outcomes between minimally invasive and open procedures (Jochum et al., 2020). Finally, epidural use has been linked to better survival in patients with ovarian cancer (de Oliveira et al., 2011; Tseng et al., 2018). In our hospital, we do not routinely use epidural anesthesia and analgesia during EOC open surgery because of the risk of life-threatening complications such as neurological deficits and epidural hematoma (Bos et al., 2017). Despite these limitations, our results may have an important clinical implication for EOC management if the relationship between anesthetics and oncological outcomes after cancer surgery is indeed causal.

## Conclusion

Propofol anesthesia was associated with better survival than desflurane anesthesia in open surgery for EOC. Propofol anesthesia also showed better outcomes in EOC patients with old age, high BMI, elevated CA-125 level, advanced FIGO stage, and prolonged operation and anesthesia time compared with desflurane anesthesia. In addition, patients given propofol anesthesia had significantly less postoperative recurrence and metastasis.

## Data Availability

The raw data supporting the conclusion of this article will be made available by the authors, without undue reservation.
